# Does self-reported chronic pain influence savoring of aesthetic experiences?

**DOI:** 10.1371/journal.pone.0259198

**Published:** 2021-11-12

**Authors:** Rosalie Weigand, Annika Moosmayer, Thomas Jacobsen

**Affiliations:** Experimental Psychology Unit, Humanities and Social Sciences, Helmut Schmidt University / University of the Federal Armed Forces Hamburg, Hamburg, Germany; Julius-Maximilians-Universität Würzburg, GERMANY

## Abstract

**Background:**

Aesthetic experiences elicit a wide range of positive emotions and have a positive impact on various health outcomes. In this context, savoring refers to a cognitive form of emotion regulation used to maintain and extend positive emotional experiences and is considered to contribute to health and well-being. Chronic pain has been linked to reduced reward-seeking behavior. This is the first study to investigate the relationship between self-reported chronic pain and savoring.

**Methods:**

We conducted an anonymous cross-sectional survey in a large non-clinical sample (opera, theater, and cabaret visitors; *n* = 322). The variables were assessed with a two-item-questionnaire.

**Results:**

Self-reported chronic pain was significantly negatively correlated with savoring (r = -.547)

**Conclusion:**

Altogether, this result helps to develop a better understanding of the effects of chronic pain in humans and to shed light on state-dependent differences in aesthetic experiences.

## Introduction

Aesthetic experiences, in particular the experience of beauty or pleasure, elicit a wide range of positive emotions [1] and have a positive impact on various health outcomes [2, 3]. In this context, *savoring* has been referred to as a cognitive form of emotion regulation used to maintain and extend positive experiences [4]. Savoring has been described as a “time-tested model of aesthetic emotion” [5] (p. 1) that refers to the appreciation and extensive processing of personal emotional information in aesthetic contexts. Savoring involves a clear focus on the experience, and has been shown to contribute to well-being, a reduction of negative affect and depression [6, 7] as well as more positive physical health outcomes [8].

It is reasonable to assume that people differ in their openness to aesthetic experience depending on their needs at a given moment. Maslow’s hierarchy of needs is a study tool for human needs namely physiological, safety, belongingness, self-esteem, and self-actualization [9, 10]. The model’s premise is that unless basic needs have been met, higher need levels are of no relevance [11]. Aesthetic needs are placed between the needs for self-esteem and self-actualization [12], or even treated as the highest need [13]. Following the models’ premise, if basal needs are unfulfilled, the relevance of aesthetic experiences will be affected. The relief of physical pain is considered a first order need by some authors [14], but traditionally, would belong to the second level of the pyramid.

A “pain that persists beyond normal tissue healing time, which is assumed to be 3 months” has been labeled as *chronic pain* by the International Association for the Study of Pain [15] (p. 1). 33% of adult Germans are affected by chronic pain, which severely impairs the quality of their lives [16]. For instance, recent investigations found higher levels of anhedonia–an impaired capacity to experience or anticipate pleasure–among chronic pain patients [17, 18]. However, for aesthetic experiences to occur, the ability to experience pleasure is critical [19]. For example, if opioid receptor activity and therewith the pleasure response in the reward circuits is manipulated, the pleasure derived from music is diminished [20]. Also, anhedonia has been linked to reduced activation in the ventromedial prefrontal cortex (vmPFC)–a brain region that is highly activated when experiencing beauty [21, 22]. Finally, anhedonia and savoring have been found to inversely relate [23]. Accordingly, the question arises if chronic pain and the savoring of aesthetic experiences also relate. Though anecdotal evidence might suggest otherwise, not every psychiatric condition negatively relates to the ability to have aesthetic experiences. For example, a recent investigation found a correlation between aesthetic experiences and anhedonia, but not depression [19].

This is the first study to assess the relationship between chronic pain and savoring of aesthetic experiences. Only few human studies have directly explored the relationship between chronic pain and any form of reward processing [24, 25]. Previous studies, mainly based on animal research, found evidence for a negative relationship between chronic pain and the wanting, but not the liking quality of reward processing. Wanting rather than liking seems to require cognitive capacities, such as attention or working memory [24]. A state of chronic pain is associated with cognitive impairment, especially reduced attentional capacity, processing speed, and memory [26]. Pain management captures all attentional resources [27], which points to a conflict with other tasks that require attention. In this context, the assessment of aesthetic experience is an important aspect to consider. A lot of research in this field has focused on how much people liked or disliked an artwork [28–30]—not considering the breadth of emotions felt in response to art [31] (but see [1]). While *liking* is associated with fluent processing and does not require much attentional capacity [32], during the process of *savoring*, persons typically are in a state of intense attention engagement [33, 34]. As attention becomes interrupted or shifts, the intensity of the experience fades [35]. So, in contrast to pleasure ratings, savoring ratings are assumed to capture even more attentional resources. For example, savoring of aesthetic experiences has been shown to be reduced when people are distracted by everyday tasks [36]. Thus, it may also be prone to interference with pain-related attentional processes. Supporting this, positive affect has been shown to be associated with lower levels of chronic pain in patients with osteoarthritis or fibromyalgia [37]. Moreover, recent evidence suggests a link between pain severity and savoring as a key means of inducing absorptive experiences [38].

In the present study, we conducted a low-threshold first investigation in a non-clinical sample to investigate whether self-reported chronic pain and the savoring of aesthetic experiences are related. We hypothesized a negative relationship between self-reported chronic pain and savoring.

## Materials and methods

### Sample

The sample was a convenience sample of 322 visitors of three German houses of music and performing arts: 33.5% were recruited in an opera house, 43.8% in a theater, and 22.7% in a cabaret house (S1 Table displays a data collection overview). Seven additional participants had to be excluded due to missing data.

Since we aimed at conducting a low-threshold investigation, participants remained anonymous. Prior to the study, participants were informed that neither gender nor age nor any other personal information would be collected. The study was explained verbally, and the participants were given the opportunity to ask questions. Then, their verbal consent to participate in the study under these circumstances was obtained. Since we did not collect any personal information, written consent was not obtained. The study received human subjects research ethics approval by a university institutional review board committee.

### Materials

To minimize the demand on participants, we assessed our variables with only one item, respectively. Savoring was measured using the question “I savored today’s show” [“Während der heutigen Vorführung habe ich den Augenblick ausgekostet”], which was answered on a 7-point rating scale ranging from 1 = *Not at all* to 7 = *Very much*. The item was adopted from a German study on savoring [39]. From the three items used in the study, we selected this one because it was the only item that included the term “savor” and therefore was considered to have the highest face validity. Also, the correlation between this item and the other items was high (*r* = .65 [36]). Since savoring is considered a unidimensional construct [39], the measurement via a single item can be considered valid.

To assess chronic pain, we used one item of the chronic pain grade questionnaire (CPG) [40–42] which is considered a reliable and valid measure for evaluation of chronic pain in both the general population and the primary health care setting [43].

The item “In the past 6 months, on average, how intense was your pain?” [“Wie würden Sie Schmerzen, die Sie gegebenenfalls in den vergangenen 6 Monaten hatten, im Durchschnitt beschreiben?”] was answered on an 11-point rating scale ranging from 0 = *No pain* to 10 = *Pain as bad as it could be*. The correlation between this individual item and the total CPG score has been reported as *r* = 0.77, which suggests sufficient reliability [44]. Regarding the issue of validity, factor analysis for the CPG scale revealed one relevant factor on which the item selected for the present study loaded (*r* = 0.83). Since, as a rule of thumb, 0.7 or higher factor loading represents sufficient variance extracted from the variable by the factor [45], this indicates that our item might be a valid representation of the underlying factor structure of the CPG.

### Design and procedure

The research design of this study was non-experimental and correlational as it studied the relationship between chronic pain and aesthetic savoring. The control variables in this study were performance house (opera, theater, cabaret) as well as sequence and polarity of the items.

In order to limit potential stimulus-specific effects based on (the content of) a specific show, we collected data in three different houses, resulting in a total of ten different shows. To attract the visitors, we used an 850mm x 2000mm banner with the company logo of the university. After the show, we asked visitors to complete a paper and pencil questionnaire consisting of two items. Item sequence and polarity of the rating scales were counterbalanced. Participants were either approached via a general introduction speech in front of a large number of visitors, or–in order to avoid self-selection—were directly approached by our team consisting of two men and two women. We alternately approached men and women, as well as younger and older persons.

### Statistical analyses

Data were analysed using IBM Statistics SPSS for Mac, version 25 (IBM Corp., Armonk, NY, USA). We performed a hierarchical regression analysis on the data with savoring as the criterion. In the first block, all dummy-coded control variables (i.e., sequence, polarity, house) were included in the regression model. In the second block, chronic pain was also included. The stepwise procedure was chosen to determine how much additional variance could be explained by the predictor variable.

## Results

In sum, 20.2% of our participants reported moderate pain (CPG = 5–7), 7.8% reported severe pain (CPG = 8–10). This percentage is slightly lower than the prevalence of chronic pain in the German population [16]. Table 1 presents means, standard deviations and the study correlation. Results of the Pearson correlation indicated that there was a significant negative correlation between self-reported chronic pain and savoring of aesthetic experiences (*r* = -.547, *p* < .001).

**Table 1:** Means, standard deviations and correlation.

Variable	*M*	*SD*	*r*
Savoring	5.47	1.57	
Chronic pain	3.53	2.28	-.547**

*Note*. *N* = 322; *M* and *SD* represent mean and standard deviation, respectively.

*p < .05

**p < .01.

Prior to conducing the hierarchical multiple regression, the relevant assumptions of this statistical analysis were tested. The assumption of singularity was met as the predictor and control variables were not a combination of other predictor and control variables. An examination of correlations revealed that no predictor or control variables were highly correlated. The histogram of standardized residuals indicated that the data contained approximately normally distributed errors, as did the normal P-P plot of standardized residuals, which showed points that were very close to the line. The scatterplot of standardized residuals showed that the data met the assumptions of homogeneity of variance and linearity.

**Fig 1:**
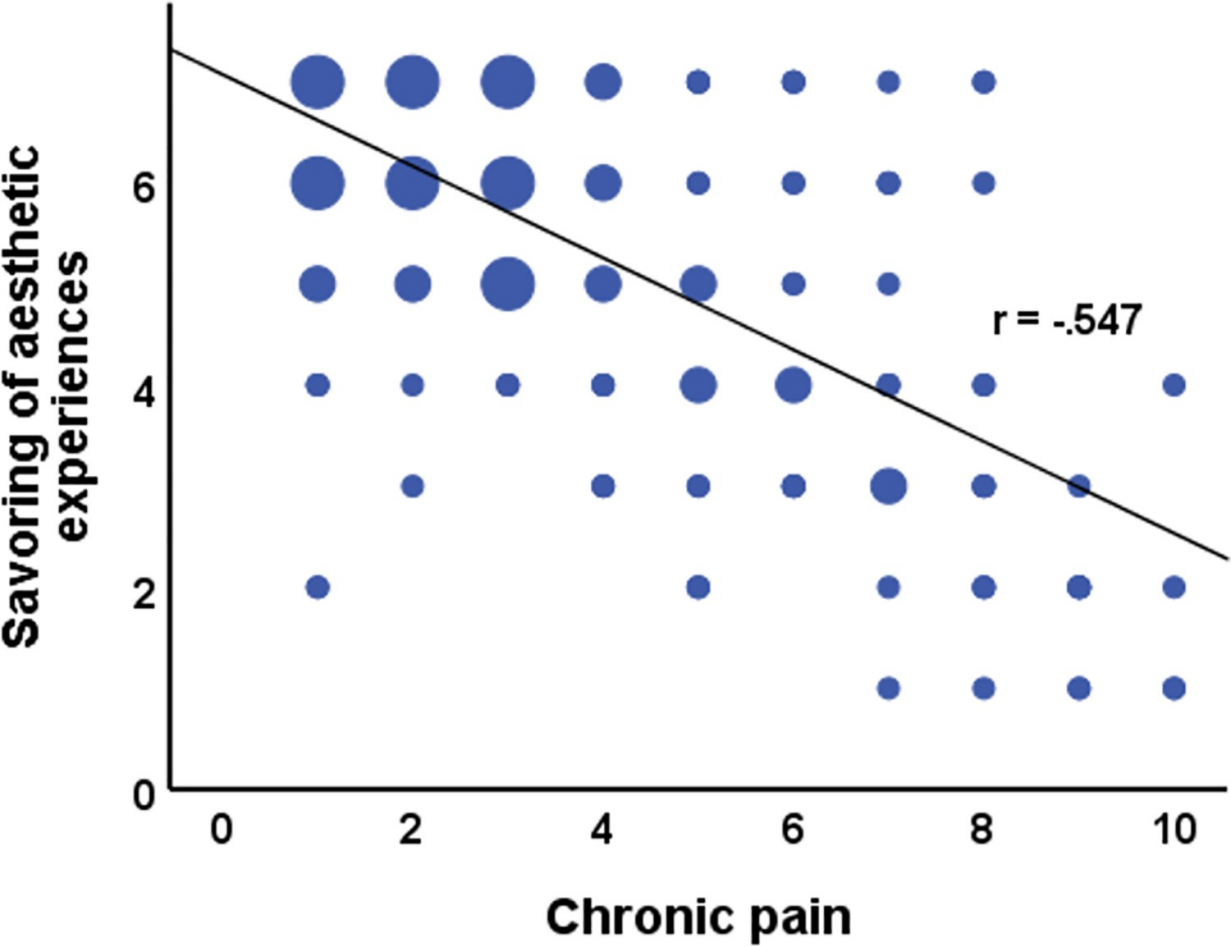
Savoring ratings by chronic pain (*N* = 322). Dot sizes indicate subsample sizes with 1–5 participants (small dots), 6–15 participants (medium dots), >15 participants (large dots).

Table 2 presents the results of the hierarchical regression analysis. The regression analysis revealed that at stage one, the control variables of opera, polarity, and sequence did not contribute significantly to the regression model. Only the effect of the dummy variable “theater” on savoring was significant, indicating that savoring was slightly reduced after theater performances compared with opera, or cabaret. Overall, the control variables accounted for only 1,8% of the variation in savoring. Introducing self-reported chronic pain explained an additional 44.5% of variation in savoring and this change in R^2^ was significant, *F* (1,316) = 243.74, *p* < .001, indicating that people with higher self-reported chronic pain savored less during the aesthetic experience (see Fig 1).

**Table 2:** Results of hierarchical regression analysis using savoring as the criterion.

Variable	Step 1 (control variables)			Step 2		
	*B*	*SE B*	*β*	*B*	*SE B*	*β*
Sequence	.050	.176	.016	.057	.132	.018
Polarity	.113	.177	.036	-.049	.133	-.016
Opera	-.114	.238	-.034	-.073	.179	-.022
Theatre	-.462	.226	-.146	-.381	.170	-.120
Pain	-	-	-	-.453	.029	-.656
R^2^	-	.018	-		.445	-
F for change in R^2^	-	1.43	-		243.74**	-

*Note*. *N* = 322

* *p* < .05

**p < .01.

## Discussion

We examined the relationship between self-reported chronic pain and savoring of aesthetic experiences in a large sample of opera, theater, and cabaret visitors. Our results indicate that higher levels of chronic pain were associated with less savoring of aesthetic experiences.

We argued that savoring involves not just the awareness of liking or pleasure, but also conscious attention to the pleasure, leading to an attentional tradeoff in chronic pain patients. Our findings of a strong negative relationship between chronic pain self-reports and savoring underpin this explanation. Also, if we can find this relationship in a non-clinical sample, it indicates that the findings could be generalized to individuals with diagnosed chronic pain.

Even though we can’t infer causality from our findings, we can insinuate the direction of the relationship based on the constructs of investigation: Chronic pain refers to a period that started in the past and participants were explicitly asked to refer to the last six months, whereas savoring is a momentary experience. It seems unlikely that the savoring experience of one evening influenced the felt average of a six-month period of pain. Thus, we interpret our results as a strong hint that chronic pain negatively influences aesthetic savoring. Since we conducted the study in a real-life setting, the findings might be generalizable to naturalistic situations. The context of aesthetic experiences affords an ecological valid study design, at some point: There is evidence that artworks presented in a museum are liked more and rated more interesting than in the laboratory [46], leaving the question open whether aesthetic savoring would be possible in the laboratory.

The results of this study also have practical implications: They suggest that people suffering from chronic pain might benefit less from positive–or more specifically: aesthetic–experiences. Since experiences of positive emotions are known to promote a variety of personal resources, such as resilience [47], it may be of relevance if people suffering from chronic pain weren’t able to fully benefit from that. An important goal of chronic pain treatment lies in the improvement of patients’ quality of life [48]. Adults who engage in more savoring report higher well-being and lower ill-being [23, 49]. Therefore, if chronic pain leads to less savoring, this could onset a downward spiral, for positive experiences being less intense and less relevant for a population that really needs them. This reduction in savoring might even form part of a vicious circle for chronic pain patients, in which a lack of savoring of positive experiences results in exacerbated pain, leading to less savoring. In this vein, our results stress the importance of integrating methods to strengthen patients’ savoring capacity into chronic pain therapy in order to improve patients’ pain management and well-being, and to break the vicious circle. For example, mindfulness has been described as a practice of learning to focus attention on momentary experiences and is becoming increasingly popular as complementary therapeutic strategy for a variety of medical and psychiatric conditions [50]. Alternatively, since liking requires less attentional capacity and doesn’t seem to be impaired in chronic pain, interventions could focus on this aspect of aesthetic experience in order to improve patients’ quality of life. This could be done using aesthetic preferences based on mere exposure or fluent processing, prototypes, attitudes, or episodic memory [51].

In addition to the implications for chronic pain treatment, our results also help to shed light on the necessary conditions for aesthetic experiences. While many studies in aesthetics have focused on objective features of stimuli [52–54] or situational aspects [55, 56] that influence aesthetic experiences, surprisingly little is known about the effects of different inner states on aesthetic experiences.

Our results suggest that when in a state of chronic pain, aesthetic experiences are hampered. On a more general level, people whose basic needs are not fulfilled might be in a motivational state associated with a bias in information processing, such as selective attention for stimuli relevant to that motivational state [57]. Therefore, our results might suggest that not only in pain, but also if other basic needs are not fulfilled, the attention-demanding savoring-quality of aesthetic experiences could be impaired. Conclusively, a necessary condition for savoring aesthetic experiences could lie in the availability of enough attentional capacities and a state of homeostatic balance in terms of the fulfilment of basic needs.

The present results highlight several areas that should be further examined to better understand their theoretical and clinical implications. Further studies are needed to examine the relationship between other basic needs and savoring of aesthetic experiences, in order to strengthen our claim regarding the necessary conditions for aesthetic savoring. Additionally, if further research clarifies that in contrast to savoring, pleasure ratings are indeed unaffected by chronic pain—as implied in the animal model—the focus should be on improving sensory based interventions (e.g., aromatherapy, massages, etc.) to improve chronic pain patients’ quality of live.

Some study limitations merit comment. The first limitation concerns the correlational nature of our data: We didn’t manipulate our predictor chronic pain and didn’t control extraneous variables. Therefore, we are not able to rule out other possible explanations for the relationship between chronic pain and savoring. We chose the correlational method because it isn’t possible to manipulate chronic pain. Experimental studies could only induce acute pain and investigate its effect on savoring. However, results between studies of acute and chronic pain might not be comparable: It is proposed that changes in the function and structure of the brain’s reward network are involved in the pathophysiology of chronic pain [58, 59], but not in acute pain. For example, in experimental studies inducing acute pain in humans, motivation to obtain reward was increased [25], whereas chronic pain seems to negatively impact this wanting quality [24]. The second limitation to consider is our measurement of chronic pain. The EHO’s International Classification of Functioning Disability and Health (ICF) identifies three main outcomes for any given health condition: Impairment, activity limitations, and participation restrictions. Our item only measures impairment [60], thereby not doing justice to the complexity of the construct. Ideally, standardized instruments for the assessment of chronic pain should not replace clinical interviews, but rather complement them [61]. Given the multidimensional nature of chronic pain, the assessment requires a multiaxial approach.

We also acknowledge that, since participants only reported two ratings, any relation between the two measures may also be affected by response biases in terms of people’s propensity to report extreme values. Therefore, it is possible that the reported effect exaggerates the true correlation between savoring and chronic pain. Additionally, we acknowledge that one-item measurement of any construct is less reliable that the use of a validated full questionnaire, even though we explained why we deemed our items suitable for assessing the constructs in question. Also, in the literature on self-report measurements, there is a recurring debate whether failure-oriented self-reports measure a global “complaints tendency” rather than the intended trait [62, 63]. According to this, it might be possible that higher ratings of chronic pain reflected participants’ individual complaints tendency rather than their actual pain. We do not know to what degree–if at all–our ratings reflect this response bias. However, since it would be reasonable to assume that this complaints tendency would also be associated with less reported savoring, the true correlation between chronic pain and savoring might be lower than reported here.

Third, we must take into account that visitors of cultural events, especially the opera, often display specific demographic characteristics. For example, according to an investigation in German cultural institutions [64], opera visitors often are of older age and possess higher education that the general population. Only 13% of opera-goers and 17% of theater-goers were 34 or younger. Even though we explicitly tried to approach older *and* younger persons, we did not assess the sample’s level of education. Accordingly, the correlation reported here might be specific to educated middle-class.

A final limitation concerns the collection of data via self-report. We chose this method of data collection to provide a low-threshold first investigation in this research area and to reach a large sample. In future work, it may be critical to include other more objective forms of data collection in order to triangulate the psychological information collected.

## Conclusions

In conclusion, this study provides evidence that chronic pain and the attention-demanding savoring quality of aesthetic experiences are inversely related, an idea that was unexplored before. Our results do not only help to develop a better understanding of the effects of chronic pain in humans, but also hint to the problem when reduced savoring of positive experiences and pain mutually reinforce each other and impair chronic pain patient’s quality of life. Consequently, our results help to point out some starting points for clinical interventions. They also help to shed light on state-dependent differences in aesthetic experiences.

## Supporting information

S1 Table Data collection overview. (DOCX)
