# Supplementary material for: Does self-reported chronic pain influence savoring of aesthetic experiences?
Source: PLoS One. 2021 Nov 12;16(11):e0259198. doi: 10.1371/journal.pone.0259198 (PMC8589147; doi:10.1371/journal.pone.0259198)
Supplement: S1 Table — (DOCX) [file pone.0259198.s001.docx]

| Table S1  *Data collection overview* | | |
| --- | --- | --- |
| House | Show (Date) | *n* |
| Staatsoper Hamburg | Le Nozze di Figaro (05.11.19) | 34 |
| Staatsoper Hamburg | Don Giovanni (06.11.19) | 33 |
| Staatsoper Hamburg | Hansel and Gretel (27.11.19) | 41 |
| Thalia-Theater | Moby Dick (13.11.19) | 29 |
| Thalia-Theater | Liliom (22.11.19) | 33 |
| Thalia-Theater | Neverland (24.11.19) | 22 |
| Thalia-Theater | The Cat and the General (28.11.19) | 39 |
| Thalia-Theater | The Crucible (07.12.19) | 18 |
| Alma-Hoppe-Lustspielhaus | Always Trouble with the Neighbors (14.11.19) | 25 |
| Alma-Hoppe-Lustspielhaus | Ausnahmezustand (21.11.19) | 24 |
| Alma-Hoppe-Lustspielhaus | Always Trouble with the Neighbors (25.11.19) | 24 |
| *Note. N* = 322 |  |  |
